# Factors Influencing Vertical Transmission of Psittacine Bornavirus in Cockatiels (*Nymphicus hollandicus*)

**DOI:** 10.3390/v14122721

**Published:** 2022-12-06

**Authors:** Jessica Link, Sibylle Herzog, Anna Maria Gartner, Bianca Bücking, Matthias König, Michael Lierz

**Affiliations:** 1 Clinic for Birds, Reptiles, Amphibians and Fish, Justus Liebig University Giessen, Frankfurter Str. 114, 35392 Giessen, Germany; 2 Institute of Virology, Justus Liebig University Giessen, Schubertstr. 81, 35392 Giessen, Germany

**Keywords:** avian bornavirus, Bornaviridae, psittaciform 1 orthobornavirus, proventricular dilatation disease, neurological disease, virus transmission, age dependency, immunopathogenesis, species conservation, vertical transmission

## Abstract

The transmission of parrot bornavirus is still not fully understood. Although horizontal transmission through wounds can be one route, vertical transmission is still discussed. PaBV RNA and PaBV antigen were detected in psittacine embryos, but isolation of the virus failed, raising doubts about this route. In this study, cockatiels were infected either as adults (adult group) or during the first 6 days after hatching (juvenile group) and raised until sexual maturity to breed and lay eggs. A total of 92 eggs (adult group: 49, juvenile group: 43) were laid and incubated until day 17. The embryos and yolk samples were examined by RT-PCR for PaBV RNA and by infectivity assay for infectious virus. In the adult group, 14/31 embryos (45.2%) and 20/39 (51%) of the yolk samples demonstrated PaBV RNA in the PCR. Isolation of PaBV was not possible in any embryo of this group, but it was achieved in six yolk samples from one female. Anti-PaBV antibodies were detected in the yolk samples after seroconversion of all female parents. In the juvenile group, 22/29 embryos (74.9%) were positive for PaBV RNA. In 9/21 embryos (42.9%), PaBV isolation was possible. PaBV RNA was detected in 100% and infectious virus in 41% of the yolk samples. Anti-PaBV antibodies were detected in all yolk samples. For the first time, successful vertical transmission of PaBV was proven, but it seems to depend on the age when the parent birds are infected. Therefore, the age of the bird at time of infection may be an important factor in the occurrence of vertical transmission.

## 1. Introduction

Psittacines are one of the most endangered avian groups. Many species are nearly extinct according to the International Union for Conservation of Nature (IUCN). One of the most threatening infectious diseases in parrots is caused by parrot bornaviruses (PaBVs), which were first detected in 2008 [1,2]. PaBVs are the causative agent of the proventricular dilatation disease (PDD). Impaired gastro-intestinal function and symptoms affecting the central nervous system may appear as clinical signs [3]. 

Different infection trials suggested PaBVs as the causative agent for PDD, as birds developed clinical signs after being infected with various virus isolates [4,5]. Piepenbring et al. [6] fulfilled the Henle–Koch postulates for PaBVs by infecting nine cockatiels through intraveneous and intracerebral routes. All those studies had in common the use of parenteral infection routes. As a natural route of transmission, a faeco-oral route was considered [7]. However, the low prevalence of PaBV infections in parrot flocks raised doubts, and Heckmann et al. [8] excluded oral and nasal inoculation as a potent infection route, which was also supported by another study [9]. Later, they demonstrated that PaBVs can probably be transmitted through wounds [10], which was supported by another study proving viral spread along peripheral nerves [11]. Apart from horizontal transmission, vertical transmission of PaBVs is discussed, as PaBV RNA was detected in 2 out of 30 embryos in one study [12]. Monaco et al. [13] demonstrated PaBV RNA in embryos of PaBV-positive parents in 10 of 61 eggs. Virus isolation was not performed. In another study, fertilized eggs of four sun conures were examined and PaBV RNA found in all eggs at the beginning of development. In late-stage embryos, PaBV was also detected in the liver, brain and eyes of the embryos. Surprisingly, after hatching, only a few chicks shed PaBV-RNA. Virus isolation was also not performed [14].

To examine vertical transmission, cockatiel embryos were inoculated with PaBV-2 or PaBV-4 [15]. Inoculation was undertaken in the yolk sac or on the chorioallantoic membrane between days 3 and 5 of egg incubation and the embryo harvested at day 17 of incubation. Depending on the route of inoculation and the virus isolate, between 90% and 100% of the embryos demonstrated PaBV-4 and PaBV-2 RNA and PaBV-4 and PaBV-2 antigens in the brain only. However, virus isolation failed. All these studies point towards vertical transmission as an option for the spread of PaBVs. However, an abortive infection may also occur and prevent vertical transmission. Last but not least, inoculation of embryonated eggs does not correspond to the natural route of infection. Additionally, a previous study demonstrated that the age of a bird at time of infection with PaBV-4 plays a role in the development of the disease and, most likely, in the epizootiology of the infection. This may depend on the full development of the immune system at time of infection [16] and may play a role in vertical transmission. Gartner et al. [16] inoculated cockatiels with PaBV-4 either in the first week after hatching or as adults. Both age groups were raised to sexual maturity and allowed to lay eggs, which served as samples for the present study. Eleven adult cockatiels and eleven cockatiels at the age of 1–6 days were inoculated intravenously with PaBV-4. Afterwards, the birds were monitored for 233 days and the course of infection was documented from the shedding of RNA (RT-PCR) and through determination of PaBV-4 antibodies by IIFA. All 22 cockatiels excreted PaBV-4 RNA and seroconverted during the experimental period. The juvenile birds demonstrated earlier RNA excretion and seroconversion than the adults. Among the adults, 9 of 11 birds exhibited clinical signs of PDD, and only one of the juvenile birds sickened and died but not as a result of PaBV infection. All others remained clinically healthy. In necropsy, 7 of 11 adult birds showed PDD compared to none in the juvenile group. The results provide strong evidence that the age of the cockatiels at the time of infection plays an important role in the expression of clinical signs [16]. The juvenile birds were not yet immunocompetent at hatching, but immunotolerance was not developed. It could be assumed that an earlier infection (such as after vertical infection) would show even larger differences compared to adult infected birds. However, it remains unclear if the findings of Gartner et al. [16] also show an effect on virus transmission routes; in particular, vertical transmission. 

Therefore, the laid eggs of the adult and juvenile infected cockatiels (after sexual maturity) from the study by Gartner et al. [16] were investigated to explore the occurrence of PaBV and anti-PaBV antibodies in the embryos and yolk at day 17 of incubation and, especially, to evaluate the influence of the age of the parent bird at time of infection on vertical transmission.

## 2. Materials and Methods

Parent cockatiels: The cockatiels that laid the eggs for this study were already part of another study [16]. The cockatiels came from an SPF flock that tested negatively for various pathogens, including paramyxovirus-1, psittacine herpesvirus, Chlamydia psittaci and PaBV. Five adult pairs and one single female were kept in cages (162.5 × 50 × 50 cm) with two nesting boxes outside. The ages of the birds were between 405 and 2142 days. Food and water cups were accessible from outside so that the breeding pairs were not disturbed. Additionally, UV light-bulbs and additional protein-rich semi-ripe millet were provided. The pairs were synchronized in breeding and, when 11 cockatiel chicks between 1 and 6 days of age were present, the 11 adult birds and the 11 chicks were inoculated intravenously with a PaBV-4 isolate at the same time. For inoculation, the PaBV-4 isolate Ps34 originated from the brain material of a scarlet macaw (*Ara macao*). The virus isolate had been used in previous studies [6,8,16].

The juveniles were named J1 to J11, whereas the adult pairs were named A to E, with 1.0 for males and 0.1 for females (Table 1). 

After weaning, the juvenile cockatiels were separated from the adult birds. The juveniles were kept either in pairs or in groups of four birds in similar cages as the adults. One cage had a group of four birds consisting of one male (J5) and three females (J3, J4 and J7). The male J5 was mated to all three female birds (J3, J4 and J7). Therefore, they were referred to as Pair 1 (J3/J5), Pair 2 (J4/J5) and Pair 3 (J7/J5). In another cage, the two birds of Pair 4 (J11/J10) were kept together (Table 2).

Both age groups were allowed to lay eggs; the juvenile group started later, after becoming sexually mature. 

To achieve a better understanding, the birds infected as chicks were called “juveniles” during the whole study. During the study, a total of 92 eggs were laid. The cockatiels infected as adults laid 49 eggs, whereas the birds infected as juveniles laid 43 eggs. D0.1 of the adult group and J1, J2, J6, J8 and J9 of the juvenile group were not breeding during the time of the study.

Experimental design: All eggs were collected and incubated at 37.4 °C and 60% humidity (HEKA-Format Brutmaschine, HEKA-Brutgeräte–Christa Hemel, Rietberg). After 17 days of incubation, the fertilized eggs were snap frozen for 10 min. Unfertilized eggs were opened and egg yolk and egg white were collected. The embryos of the fertilized eggs were decapitated and the pooled organs (liver, kidneys, gastrointestinal tract, heart), brain, retina and yolk were sampled for further examination by real-time RT-PCR to detect PaBV-4 RNA. Tissue from the brain, retina, pooled organs (liver, kidney, heart and gastrointestinal tract) and yolk were used for virus isolation in an infectivity assay. Embryos that died during incubation were examined as total bodies, if there was not enough material for necropsy, using PCR and virus isolation. 

Egg yolk of unfertilized eggs was tested for PaBV-4 RNA, infectious virus and anti-PaBV antibodies, respectively.

PaBV-specific real time RT-PCR: To extract RNA from the samples, the RNeasy Mini Kit (Qiagen, Hilden, Germany) was used according to the manufacturer’s instructions. Afterwards, the RNA of each sample was transcribed by random hexamer primers (Qiagen, Hilden, Germany) according to the manufacturer’s instructions. A Rotor-Gene Q cycler (Qiagen, Hilden, Germany) was used to perform the real-time RT-PCR. The real-time RT-PCR was prepared following Honkavuori et al. [2] using the primer 1034–1322.

Virus isolation: For virus isolation, an infectivity assay was performed as described previously [17]. Briefly, tissue samples and yolk were collected in Gibco TM Glasgow Minimum Essential Medium (Thermo Fisher Scientific, Walthem, MA, USA) with 2% fetal bovine serum. Tissues were suspended in medium (10%, wt/vol) and yolk (50%, wt/vol), sonicated, clarified by centrifugation (1000× *g* for 10 min) and incubated with CEC-32 cells for six days at 37 °C. Virus replication was visualized with an indirect immunofluorescence assay (IIFA) using a polyclonal serum from experimentally BoDV-infected rats [18]. 

Serology: Egg yolk samples were tested using an indirect immunofluorescence assay (IIFA) for antibodies against PaBVs as previously described [18]. Yolk samples were diluted 1:2 in PBS def., frozen once and clarified by centrifugation (2000× *g* for 5 min). The supernatant was tested for antibodies against PaBVs. Briefly, the supernatant of the yolk samples was incubated on slides with acetone-fixed CEC-32 cells persistently infected with the PaBV isolate Ps34. After incubation for 30 min, cells were exposed for 30 min to fluorescein isothiocyanate (FITC)-conjugated goat anti-bird IgG (Bethyl Lab., Inc. Montgomery, TX, USA). Yolk samples were diluted twofold and titrated to the end point [18].

## 3. Results

### 3.1. Eggs

During the time of observation (233 days), 92 eggs were laid in total. The adult group laid 49 eggs, with 31 being fertilized and 18 unfertile (Table 3). The first egg was laid 38 days after infection. 

In the juvenile group, 43 eggs were laid, 28 of which were fertilized and 15 unfertilized (Table 4). Here, the first egg was laid 160 days after inoculation, as the cockatiels had to mature first. The females of both groups started shedding PaBV-4 RNA consistently via the cloaca prior to the first egg being laid. 

Pair 1 (A0.1/A1.0) only laid one egg because the female bird (A0.1) died early during the study with unspecific symptoms, such as weight loss and apathy. Pair 2 (B0.1/B1.0) laid four eggs. Three of them were fertilized. Pair 3 (C0.1/C1.0) laid 31 eggs in total: 27 were fertilized and 4 were unfertilized. Pair 4 (E0.1/D1.0) had ten unfertilized eggs. Pair 5 (F0.1/E1.0) laid three eggs, also all unfertilized. 

In the juvenile group, the four birds J3, J4, J5 and J7 were held together. The male bird J5 was breeding with three different females (J3, J4 and J7). Therefore, they were designated Pair 1 (J3/J5), Pair 2 (J4/J5) and Pair 3 (J7/J5). There were two nesting boxes per cage. Pair 1 (J3/J5) was breeding in the same nesting box with another female (Pair 2: J4/J5). The female bird J4 started laying eggs first, so a few eggs could be identified as hers. After a few days, female bird J3 also started laying eggs in the same nesting box. Therefore, the eggs could no longer be kept apart and were named J3/4. The male bird of Pairs 1 and 2 also fertilized eggs with another bird (Pair 3: J7/J5), which could be kept separate from the eggs of Pairs 1 and 2 because they had a separate nest in the other nesting box of the cage. Pair 4 consisted of two birds (J11/J10) that were kept in a separate cage. Pairs 1 and 2 laid 31 eggs together: 18 fertilized and 13 unfertilized. Ten embryos were dead before reaching day 17 in the incubator. Pair 2 laid three eggs that could be kept apart: one was fertilized, two were unfertilized. Pair 3 laid one fertilized egg which was the only egg that was laid in total. This one embryo was dead before day 17. Pair 4 laid eight fertilized eggs, and all embryos survived until day 17. 

### 3.2. PCR

#### 3.2.1. Embryos

Adult group: In 15/31 (48%) of the embryos (Appendix A), PaBV-4 RNA was detected. The one fertile egg of Pair 1 (A0.1/A1.0) and all fertile eggs of Pair 2 (B0.1/B1.0) were negative in the PaBV-specific RT-PCR. In Pair 3, 15 of 27 (56%) embryos were positive in the PaBV-specific RT-PCR, the first being positive 141 days after infection of the female parent. Pairs 4 and 5 did not have any fertile eggs.

Juvenile group: In the juvenile group, 25/28 (89.3%) embryos (Appendix B) were positive in the PaBV-specific RT-PCR, and 16/18 (89%) embryos of Pairs 1 and 2 were positive in the PCR. The embryo of Pair 3 was also positive in the PCR (1/1, 100%). All fertile eggs (8/8, 100%) of Pair 4 were positive in the PCR. 

#### 3.2.2. Egg Yolk

Adult group: In 20/39 (51%) egg yolks in the adult group, PaBV-4 RNA was detected, and 18/22 (82%) egg yolks of Pair 3 and 2/10 (20%) of Pair 4 were positive in the PaBV-4 PCR. All three eggs of Pair 5 in the adult group were negative for the detection of PaBV-4 RNA.

Juvenile group: In the juvenile group, 27/27 (100%) egg yolks tested positive for PaBV-4 RNA. All 23/23 (100%) of the yolks of Pairs 1 and 2 of the juvenile group were positive in the PCR. The yolk of the one egg of Pair 3 was not examined by PaBV-4 PCR. All egg yolks of Pair 4 (4/4, 100%) tested positive for PaBV-4 RNA.

### 3.3. Virus Isolation

#### 3.3.1. Embryos

**Figure 1:**
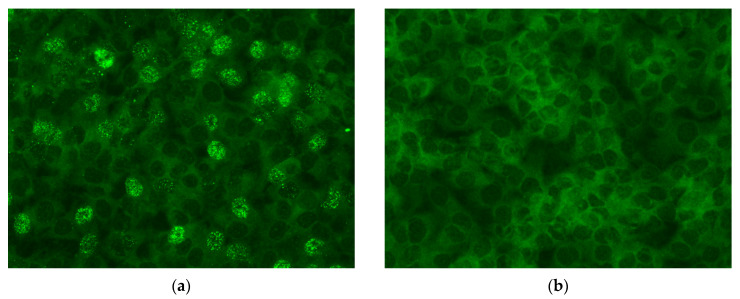

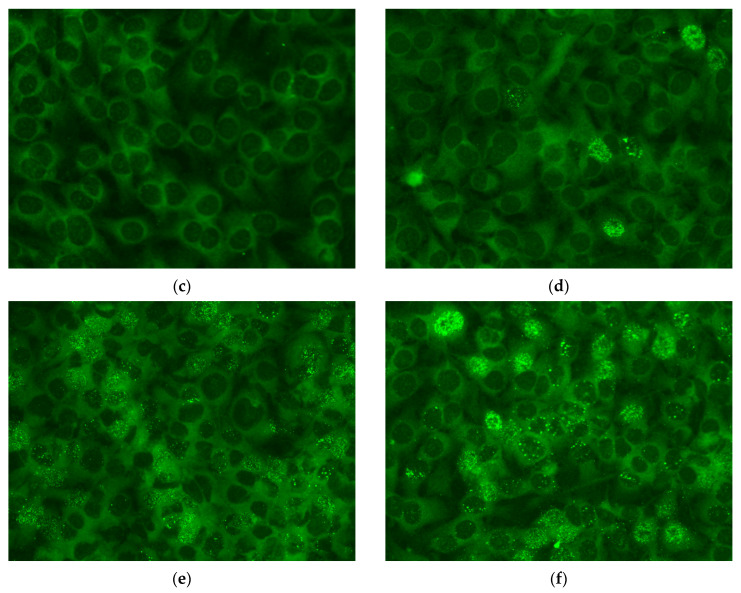
(**a**) Infectivity assay: CEC-32 cell culture with immunofluorescence of PaBV-specific antigen—juvenile group, yolk sample J11/54 (positive), 200× total magnification; (**b**) infectivity assay: CEC-32 cell culture with immunofluorescence of PaBV-specific antigen—juvenile group, yolk sample J3/4/55 (negative), 200× total magnification; (**c**) infectivity assay: CEC-32 cell culture with immunofluorescence of PaBV-specific antigen—adult group, embryo C0.1/61 (negative), 200× total magnification; (**d**) infectivity assay: CEC-32 cell culture with immunofluorescence of PaBV-specific antigen—adult group, yolk sample C0.1/53 (positive), 200× total magnification; (**e**) infectivity assay: CEC-32 cell culture with immunofluorescence of PaBV-specific antigen—juvenile group, embryo J3/4/77 (positive), 200× total magnification; (**f**) infectivity assay: CEC-32 cell culture with immunofluorescence of PaBV-specific antigen—juvenile group, brain J11/76 (positive), 200× total magnification.

Adult group: In the adult group, virus isolation failed in all 31 embryos of the different pairs (Table 3 and Figure 1c). 

Juvenile group: In the juvenile group, PaBV-4 was isolated from 9/21 (42.9%) embryos. PaBV-4 was isolated from 6/15 (40%) of the embryos from Pairs 1 and 2, 1/1 (100%) from Pair 3 and 2/5 (40%) from Pair 4. Virus isolation was also possible in the dead embryos of Pairs 1 and 2 in five out of six embryos (83%) and in the only embryo (100%) of Pair 3. Pair 4 had no dead embryos (Table 4 and Figure 1e,f).

#### 3.3.2. Yolk

Adult group: In the adult group, 6/37 (16%) egg yolks were positive in the infectivity assay, all of them originating from the fertilized eggs of Pair 3 (Table 3 and Figure 1d). PaBV-4 isolation was not performed for the yolks of Pair 1 and only in one yolk of Pair 2. With Pair 4 (ten yolks) and Pair 5 (three yolks), virus isolation failed. All those yolks came from unfertilized eggs because Pairs 4 and 5 did not have any fertilized eggs. The virus was only isolated in cases where the yolk was also positive for PaBV PCR with ct values below 30.

Juvenile group: In the juvenile group, PaBV-4 isolation was possible in 11/27 (41%) egg yolks of fertilized eggs (Table 4, Figure 1a,b). In detail, for Pair 1 and Pair 2 of the juvenile group, PaBV-4 was isolated in six out of seven (86%) of egg yolks from the fertilized eggs. One yolk was toxic for the cells and, therefore, no result was obtained. The virus could also be isolated from the only (100%) yolk from Pair 2 and from four out of five (80%) yolks of Pair 3. The yolk of one fertilized egg from Pair 4 was not examined in the infectivity assay. The unfertilized eggs in the juvenile group (12/12) were all negative in the PaBV-4 isolation.

### 3.4. Serology

**Figure 2:**
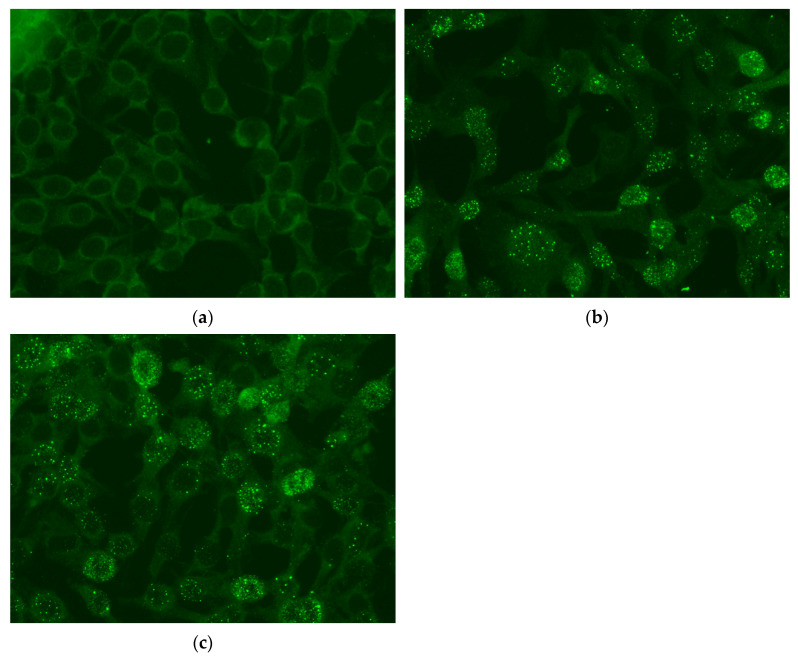
(**a**) No detection of anti-PaBV antibodies from yolk sample B0.1/4 before seroconversion (titer < 1:10) by indirect immunofluorescence assay (IIFA) on CEC-32 cells, 200× total magnification; (**b**) anti-PaBV antibody detection from yolk sample B0.1/5 after seroconversion (titer 1:640) by indirect immunofluorescence assay (IIFA) on CEC-32 cells, 200× total magnification; (**c**) anti-PaBV antibody detection from yolk sample J11/81 (titer 1:1280) by indirect immunofluorescence assay (IIFA) on CEC-32 cells, 200× total magnification.

In 45 of 49 (92%) of the egg yolks (from fertilized and unfertilized eggs) of the adult group, anti-PaBV antibodies were detected. The antibody titers ranged from 640 to 5120. The four negative eggs originated from birds A0.1 and B0.1 and were laid before seroconvertion of the female parent (Figure 2a). 

In all 43 egg yolks (from fertilized and unfertilized eggs) in the juvenile group, anti-PaBV antibodies were detected with antibody titers comparable to the adult group (Figure 2b,c).

## 4. Discussion

The aim of the study was to investigate the possibility of vertical transmission of PaBV-4 in parrots with regard to the age at time of infection of the parent birds. For the first time, vertical transmission was demonstrated, but there were differences in the prevalence of PaBV-4-positive eggs and the isolation of the infectious virus with regard to the age when the parent birds were infected with PaBV-4. Vertical transmission occurred from parent birds that were infected as nestlings and the infectious virus could only be detected in embryos under this condition. In birds infected as adults, the infectious virus was not found in embryos but only in the yolks of a low number of eggs laid from just one bird. However, PaBV-4 RNA was also detected from embryos of the adult group. 

In the adult group, isolation of PaBV-4 was only possible in 6/37 yolks of Pair 3. Interestingly, the female parent of these eggs was the youngest (405 days) in the adult group at the time of infection. It was also noticeable that this bird developed no clinical symptoms and showed no dilatation of the proventriculus after infection with PaBV-4 [16], which was comparable to the birds infected as juveniles. However, it remains unclear if the detection of PaBV-4 RNA in the embryos of the adult group was for an abortive infection or also a valid vertical transmission but with a viral load too low for virus isolation, as was also speculated by Wüst et al. [15]. Further studies must be carried out in order to evaluate this and determine whether the potential immunotolerance of the birds supports vertical transmission or not. 

In the adult group, 48% of the embryos were positive in the PaBV-4-specific RT-PCR, while the juvenile group had more than 89% positive embryos in the PaBV-specific PCR. Interestingly, all embryos of the adult group survived until they were harvested, while 8/19 embryos of the juvenile group died during incubation. These dead embryos originated from female birds that had been infected four days after hatching. PaBV-4 was isolated from four out of five dead embryos. In contrast, all eight embryos of a female bird infected one day after hatching were alive. For this bird, PaBV-4 was isolated from two out of five embryos and four out of five yolks. However, individual effects of pairing leading to embryonic death cannot be excluded. Therefore, it cannot be speculated whether the death of the embryo was triggered by PaBV-4. As none of the parent birds that were infected as juveniles during their first week of life demonstrated any clinical signs, it seems unlikely that embryos infected vertically develop clinical disease. It seems more likely that vertical transmission creates carrier birds comparable to those infected during the first week of life. This might be due to the immune incompetence at the time of infection, as PaBV-4 infections trigger an immune-mediated clinical disease [19]. However, this theory needs to be further evaluated and proven. 

Interestingly, the parent birds of both groups shed PaBV-4 RNA at the time that they laid the first eggs. Therefore, shedding of PaBV-4 RNA seems not to be correlated with vertical transmission and isolation of infectious virus from the embryos. However, the amount of PaBV-4 virus shed at the time of egg laying, as well as the tissue distribution of PaBV-4 during egg laying in individual birds, might be factors influencing vertical transmission; this was not investigated in this study but might be a focus in the future. However, as the differences between the two groups were not high [16], this does not seem likely. Additionally, the presence of anti-PaBV antibodies in the serum of the parent birds, as well as in the yolk of the eggs, did not seem to affect transmission. After seroconversion of the female parent, antibodies were detected in nearly all egg yolks examined. Only two females of the adult group (the oldest female birds) laid four eggs before seroconversion. As expected, no antibodies were detectable in those yolks. In the juvenile group, the antibodies in the yolk had no effect on virus replication in the embryos and did not protect the embryos from infection. To date, no neutralizing antibodies against PaBV have been described [20].

It should be considered that the time between infection of the parent bird and the start of egg laying varied between the groups, and this could have been another reason for the detected differences in vertical transmission rather than the age of the parent bird at time of infection. The juvenile group laid their eggs after sexual maturity, while the adults laid eggs right at the beginning and throughout the complete experimental period. The first egg of the juvenile group was laid 160 days after infection. From day 167 p.i., the first positive virus isolations of the egg yolk and positive PCR results were obtained in this group. Positive virus isolations from the embryos occurred from day 208 p.i. onwards. The eggs of the adult group were already being examined up to day 160 p.i., as well as later, and they were comparable after day 208 p.i. Here too, no positive virus isolation was detected from the embryos. However, the numbers of fertilized eggs in the adult group after 160 days p.i. were limited, and it cannot be excluded that single eggs would have been positive in virus isolation after this time point. Nevertheless, it seems unlikely that the time after infection was the main reason for the differences between the groups. Adults and juveniles started shedding PaBV from 31 days p.i. onwards in similar amounts but, in the adult group, the embryos did not test positive in the infectivity assay at any time. In addition, the differences between the two groups in the occurrence of PaBV-RNA and life virus in the yolk and embryos were also very high for infertile eggs, which were also laid in the adult group after 160 dpi in considerable numbers. Furthermore, the virus was already detected in the yolk of infertile eggs at day 141 in the adult group. Lastly, both juvenile and adult parents tested positive for PaBV-4 in all organs [16], which makes a difference in transmission due to the time span between infection and egg laying unlikely. Therefore, it seems appropriate to assume that the age of the bird at the time of infection played a larger role in the detected differences, as the epizootiology also varied between those two groups [16]. However, this should be clarified in future studies involving a larger amount of parent birds infected at different times and with the collection of more eggs at later time points. 

Detection of infectious PaBV-4 in embryos of parent birds infected shortly after hatching confirms vertical transmission for the first time. This transmission route may be responsible for the creation of carrier birds and the maintenance of PaBV-4 in the psittacine population. Those birds could potentially infect other birds through horizontal transmission through wounds [10], and birds infected in this way might develop disease, as previously shown [16]. Knowing that vertical transmission is possible, control strategies in flocks can be developed to prevent further infections. Additionally, it is possible that not all eggs of PaBV-4-infected parents were infected by vertical transmission (9/31 (29%) of the eggs from the adult group were negative in the PaBV-specific PCR and virus isolation and none from the juvenile group), although there could also have been too small an amount of the virus, insufficient for detection. In species conservation breeding programs, this information is highly important, as PaBV-infected rare parent birds might still be used as breeders since it seems to be possible to obtain negative offspring from them, which has already been speculated before [3] and also demonstrated in canary birds [21]. 

With this study, better control over PaBV infections should be possible in the future in addition to better management of breeding flocks. It seems that controlling vertical transmission might control the spread of the disease.

## Figures and Tables

**Table 1:** PaBV history of the parent birds: 0.1 is female, 1.0 is male.

				First Detection (dpi)	
Group	ID	Sex	Age at Time of Infection in Days/Years	Anti-PaBV Antibodies (Titer)	PaBV RNA
**A (adult infected)**	A0.1	Female	1814/4.9	36 (1:640)	36
	A1.0	Male	2142/5.8	57 (1:2560)	48
	B0.1	Female	668/1.8	57 (1: 320)	31
	B1.0	Male	2043/5.6	57 (1:2560)	41
	C0.1	Female	405/1.2	71 (1:320)	50
	C1.0	Male	493/1.4	50 (1:80)	41
	D0.1	Female	629/1.7	71 (1:1280)	45
	D1.0	Male	665/1.8	36 (1:40)	66
	E0.1	Female	422/12	36 (1:160)	55
	E1.0	Male	417/1.1	71 (1:5120)	50
	F0.1	Female	424/1.2	57 (1:1280)	31
**J (juvenile infected)**	J1	Male	6	50 (1:320)	36
	J2	Female	5	50 (1:320)	34
	J3	Female	4	36 (1:40)	29
	J4	Female	4	43 (1:80)	31
	J5	Male	3	43 (1:160)	27
	J6	Female	3	57 (1:1280)	27
	J7	Female	1	36 (1:80)	34
	J8	Male	1	99 (1:640)	52
	J9	Female	1	36 (1:40)	27
	J10	Male	1	43 (1:80)	29
	J11	Female	1	50 (1:640)	34

**Table 2:** Pairs of adult and juvenile infected birds. Those marked with * were nesting in a group, and the male J5 had several females.

Infected as Adults (f/m)	Age at Infection (m/f) in Days	Infected as Juveniles (f/m)	Age at Infection (m/f) in Days
Pair 1 (A0.1/A1.0)	2142/1814	Pair 1 (J3/J5 *)	4/3
Pair 2 (B0.1/B1.0)	2043/668	Pair 2 (J4/J5 *)	4/3
Pair 3 (C0.1/C1.0)	493/405	Pair 3 (J7/J5 *)	1/3
Pair 4 (E0.1/D1.0)	665/422	Pair 4 (J11/J10)	1/1
Pair 5 (F0.1/E1.0)	417/424		

**Table 3:** Adult infected cockatiels: vertical transmission. Not all embryos were examined in all tests due to technical reasons. NP: not performed.

Breeding Pair	Number of Eggs	Fertilization Fertile Infertile		PaBV-4 RT PCR Embryo		Virus Isolation Embryo		Virus Isolation Egg Yolk		Dead Embryos
				Positive	Negative	Positive	Negative	Positive	Negative	
Pair 1	1	1	0	0	1	0	1	NP	NP	0
Pair 2	4	3	0	0	3	0	3	NP	NP	0
		0	1	NP	NP	NP	NP	0	1	
Pair 3	31	27	0	15	12	0	27	6	14	1
		0	4	NP	NP	NP	NP	0	1	
Pair 4	10	0	10	NP	NP	NP	NP	0	10	0
Pair 5	3	0	3	NP	NP	NP	NP	0	3	0

**Table 4:** Juvenile infected cockatiels: vertical transmission. Pairs marked with * were nesting in a group. Therefore, it was not possible to know which birds laid which eggs. Not all embryos were examined in all tests due to technical reasons. NP: not performed.

Female Bird Group J	Number of Eggs	Fertilization Fertile Infertile		PaBV-4 RT PCR Embryo		Virus Isolation Embryo		Virus Isolation Egg Yolk		Dead Embryos
				Positive	Negative	Positive	Negative	Positive	Negative	
Pairs 1 and 2 *	31	18	0	15	3	6	9	6	1	10
		0	13	NP	NP	NP	NP	0	12	
Pair 2	3	1	0	1	0	0	1	1	0	0
		0	2	NP	NP	NP	NP	0	2	
Pair 3	1	1	0	1	0	1	0	NP	NP	1
Pair 4	8	8	0	8	0	2	3	4	1	0

## Data Availability

Not applicable.

## Appendix A

**Table A1:** Results of PaBV-specific PCR in embryo brains and egg yolk and results of virus isolation from embryo and egg yolk in the adult infected group sorted by egg number; “+” means the egg was fertilized, “−“ means the egg was unfertilized. A0.1 seroconverted at 36 dpi with a titer of 1:640, B0.1 seroconverted at 57 dpi with 1:320, C0.1 seroconverted at dpi 71 with 1:320, E0.1 seroconverted at 36 dpi with 1:160, F0.1 seroconverted at day 57 with 1:1280.

Bird/Egg No.	Egg Laid dpi	Egg Laid Days Post-Seroconversion	Anti-PaBV Titer of Egg Yolk	Fertilized	Virus Isolation Brain/Embryo	Virus Isolation Egg Yolk	PaBV PCR of Brain/ct Value	PaBV PCR of Yolk/ct Value
A0.1 1	38	2	<10	+	Negative	Not performed	Negative/−	Negative/−
B0.1 2	52	−5	<10	+	Negative	Not performed	Negative/−	Negative/−
3	54	−3	<10	+	Negative	Not performed	Negative/−	Negative/−
4	56	−1	<10	+	Negative	Not performed	Negative/−	Negative/−
5	71	14	640	−	N/A	Negative	N/A	Not performed
C0.1 6	106	35	2560	+	Negative	Not performed	Negative/−	Positive/35.27
7	107	36	2560	+	Negative	Negative	Negative/−	Not performed
8	109	38	2560	+	Negative	Not performed	Negative/−	Positive/36.59
10	111	40	2560	+	Negative	Negative	Negative/−	Negative/−
11	113	42	2560	+	Negative	Negative	Negative/−	Positive/38.23
12	122	51	1280	+	Negative	Negative	Negative/−	Positive/37.08
13	123	52	1280	+	Negative	Negative	Negative/−	Negative/−
14	129		2560	−, shrivelled	N/A	Negative	N/A	Not performed
15	125	54	2560	+	Negative	Negative	Negative/−	Negative/−
16	126	55	5120	+	Negative	Negative	Negative/−	Negative/43.46
19	141	70	2560	+	Negative	Negative	Positive/35.43	Positive/36.19
20	143	72	2560	+	Negative	Negative	Negative/−	Positive/37.19
21	145	74	1280	+	Negative	Negative	Negative/−	Positive/33.34
23	147	76	2560	+	Negative	Negative	Negative/−	Positive/30.69
27	159	88	1280	+	Negative	Positive	Positive/33.55	Not performed
28	161	90	5120	+	Negative	Negative	Positive/37.53	Positive/27.38
29	163	92	5120	+	Negative	Positive	Positive/35.90	Positive/27.10
34	165	94	5120	+	Negative	Positive	Positive/36.63	Positive/26.97
42	180	109	5120	+	Negative	Positive	Positive/35.29	Positive/28.19
44	182	111	1280	+	Negative	Positive	Positive/34.44	Positive/26.70
47	184	113	1280	+	Negative	Negative	Positive/31.36	Positive/26.24
50	186	115	1280	+	Negative	Negative	Positive/32.28	Positive/30.08
53	189	118	2560	+	Negative	Positive	Positive/34.67	Positive/25.42
61	202	131	5120	+	Negative	Not performed	Dead embryo positive/	Not performed
63	204	133	2560	+	Negative	Not performed	Positive/36.94	Not performed
65	206	135	2560	+	Negative	Negative	Positive/33.71	Positive/25.66
68	208	137	2560	+	Negative	Not performed	Positive/31.53	Not performed
69	210	139	2560	+	Negative	Not performed	Positive/30.69	Not performed
71	212	141	5120	−	N/A	Negative	N/A	Positive/28.57
85	227	156	2560	−	N/A	Not performed	N/A	Not performed
89	229	158	2560	−	N/A	Not performed	N/A	Not performed
E0.1 9	111	75	2560	−	N/A	Negative	N/A	Negative/−
17	140	104	2560	−	N/A	Negative	N/A	Negative/−
18	141	105	5120	−	N/A	Negative	N/A	Positive/33.71
22	143	107	2560	−	N/A	Negative	N/A	Negative/−
46	183	147	2560	−	N/A	Negative	N/A	Negative/44.26
48	185	149	2560	−	N/A	Negative	N/A	Negative/44.18
51	187	151	1280	−	N/A	Negative	N/A	Negative/−
78	218	182	5120	−	N/A	Negative	N/A	Negative/−
79	219	183	5120	−	N/A	Negative	N/A	Positive/33.46
82	221	185	5120	−	N/A	Negative	N/A	Negative/−
F0.1 24	156	99	5120	−	N/A	Negative	N/A	Negative/−
25	158	101	2560	−	N/A	Negative	N/A	Negative/−
26	160	103	2560	−	N/A	Negative	N/A	Negative/−

## Appendix B

**Table A2:** Results of PaBV-specific PCR in embryo brains and egg yolk and results of virus isolation from embryo and egg yolk in the adult infected group sorted by egg number; “+” means the egg was fertilized, “−“ means the egg was unfertilized. Eggs with the same dpi were laid on the same day in one nest (in nos. 86 and 90, the brain could be examined separately despite the embryo dying until day 17 in the incubator).

Bird/Egg No.	Egg Laid dpi	Egg Laid Days Post-Seroconversion	Anti-PaBV Titer of Egg Yolk	Fertilized	Virus Isolation Brain/Embryo	Virus Isolation Egg Yolk	PaBV PCR of Brain/ct Value	PaBV PCR of Yolk/ct Value
J3/4 32	164	N/A	160	−	N/A	Negative	N/A	Positive/29.87
33	164	N/A	2560	−	N/A	Negative	N/A	Positive/35.85
35	166	N/A	2560	−	N/A	Negative	N/A	Positive/34.91
36	167	N/A	1280	+	Negative	Positive	Positive/36.84	Positive/30.66
37	167	N/A	640	+	Negative	Positive	Positive/36.89	Positive/31.38
38	169	N/A	640	+	Not performed	Negative	Positive/embryo 33.68	Positive/32.37
40	178	N/A	320	+	Negative	Toxic	Negative/−	Positive/30.25
41	179	N/A	640	+	Negative	Positive	Negative/−	Positive/25.67
43	181	N/A	2560	+	Negative	Positive	Positive/33.56	Positive/28.28
45	183	N/A	1280	+	Not performed	Positive	Positive/embryo 29.77	Positive/28.13
49	185	N/A	2560	+	Not performed	Positive	Positive/embryo 30.92	Positive/28.21
55	192	N/A	640	−	N/A	Negative	N/A	Positive/26.27
58	194	N/A	1280	−	N/A	Negative	N/A	Positive/31.87
59	197	N/A	1280	−	N/A	Negative	N/A	Positive/29.12
60	201	N/A	1280	−	N/A	Negative	N/A	Positive/30.85
62	203	N/A	1280	−	N/A	Negative	N/A	Positive/30.41
64	206	N/A	1280	−	N/A	Negative	N/A	Positive/32.62
66	208	N/A	1280	+	Negative	Not performed	Negative/−	Not performed
67	208	N/A	1280	+	Positive	Not performed	Positive (embryo)/31.08	Not performed
70	210	N/A	2560	+	Negative	Not performed	Positive (embryo)/28.56	Not performed
73	215	N/A	1280	+	Negative	Not performed	Positive/33.94	Not performed
75	216	N/A	2560	+	Positive	Not performed	Positive/28.77	Not performed
77	218	N/A	2560	+	Positive	Not performed	Positive (embryo)/25.62	Not performed
83	221	N/A	1280	+	Not performed	Not performed	N/A	Not performed
84	225	N/A	1280	−	N/A	Negative	N/A	Positive/31.14
86	227	N/A	2560	+	Positive	Not performed	Positive/38.02	Not performed
87	227	N/A	2560	−	N/A	Negative	N/A	Positive/31.87
88	228	N/A	1280	−	N/A	Negative	N/A	Positive/29.49
90	229	N/A	2560	+	Positive	Not performed	Positive/29.90	Not performed
91	231	N/A	2560	+	Positive	Not performed	Positive/31.64	Not performed
92	232	N/A	2560	+	Negative	Not performed	Positive/31.86	Not performed
J4 30	160	117	5120	−	N/A	Negative	N/A	Positive/35.07
31	161	118	5120	−	N/A	Negative	N/A	Positive/31.64
39	171	128	640	+	Negative	Positive	Positive/34.20	Positive/27.69
J7 80	220	184	2560	+	Positive	Not performed	Positive (embryo)/31.29	Not performed
J11 52	189	153	1280	+	Not performed	Positive	Positive/24.24	Positive/25.62
54	191	141	640	+	Not performed	Positive	Positive/32.51	Positive/25.08
56	193	143	640	+	Not performed	Positive	Positive/32.04	Positive/20.87
57	194	144	640	+	Negative	Positive	Positive/32.36	Positive/25.60
72	214	164	1280	+	Negative	Not performed	Positive/32.94	Not performed
74	216	166	2560	+	Negative	Not performed	Positive/31.02	Not performed
76	218	168	1280	+	Positive	Not performed	Positive/30.11	Not performed
81	220	170	1280	+	Positive	Negative	Positive/28.26	Not performed
